# Sedative Potential of L (+)‐Tartaric Acid: Behavioral Evidence With In Silico Insights Into Possible GABAergic Interaction

**DOI:** 10.1002/brb3.71870

**Published:** 2026-10-28

**Authors:** Sumaya Akter Bithi, Anike Chakrabarty, Khadija Akter, Chinmoy Kumar Saha, Mohammad Y. Alshahrani, Md. Arif Hossain, Emon Mia, Noshin Tasnim Yana, Afiya Ajmee, Proma Mandal, Md. Sakib Al Hasan, Muhammad Torequl Islam

**Affiliations:** ^1^ Department of Pharmacy Gopalganj Science and Technology University Gopalganj Bangladesh; ^2^ Department of Biotechnology Faculty of Engineering and Science University of Greenwich Medway Kent UK; ^3^ Department of Pharmaceutical Technology Faculty of Pharmacy University of Asia Pacific Dhaka Bangladesh; ^4^ Central labs King Khalid University Abha Saudi Arabia; ^5^ Department of Clinical Laboratory Sciences, College of Applied Medical Sciences King Khalid University Abha Saudi Arabia; ^6^ Department of Pharmacy University of Asia Pacific Dhaka Bangladesh; ^7^ Phytochemistry and Biodiversity Research Laboratory BioLuster Research Center Ltd Gopalganj Bangladesh; ^8^ Pharmacy Discipline Khulna University Khulna Bangladesh

**Keywords:** in vivo, insomnia, latency, L (+)‐tartaric acid, sedative like effect

## Abstract

**Purpose:**

Insomnia is a prevalent neurological disorder often caused by stress, anxiety, and imbalances in the GABAergic system. Although benzodiazepines like diazepam (DZP) are commonly prescribed, their long‐term use is associated with adverse effects, including dependence and impaired coordination. Therefore, there is growing interest in natural alternatives with safer pharmacological profiles.

**Method:**

This study aimed to investigate the sedative‐like potential of L (+)‐tartaric acid (LTA), a naturally occurring organic acid, through both in vivo and in silico approaches targeting the γ‐aminobutyric acid A (GABA_A_) receptor. Sedative‐like activity was evaluated in *Swiss* albino mice using thiopental sodium‐induced sleeping time, open‐field, and rotarod tests.

**Findings:**

LTA significantly reduced sleep latency and prolonged sleep duration in a dose‐dependent manner, with the higher dose (240 mg/kg) producing a more pronounced effect. Co‐administration of LTA with DZP further enhanced thiopental‐induced hypnosis. In the open‐field test, LTA reduced locomotor and exploratory activities, as evidenced by decreases in square crossings, rearing, and grooming behaviors. Rotarod assessment demonstrated a dose‐dependent reduction in motor coordination and neuromuscular performance, although the impairment remained less pronounced than that produced by DZP at the lower tested dose. Molecular docking revealed lower binding affinity than DZP but demonstrated multiple hydrogen‐bond (HB) interactions with key amino acid (AA) residues of the GABA_A_ receptor, suggesting a potentially stable receptor interaction.

**Conclusion:**

Collectively, the behavioral and computational findings indicate that LTA possesses sedative‐like properties and may modulate GABAergic neurotransmission. Further mechanistic, pharmacokinetic, and electrophysiological studies are required to clarify its precise mode of action and therapeutic potential.

## Graphical abstract

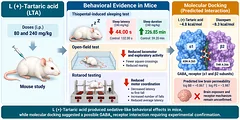

## 1 Introduction

Insomnia is a condition characterized by difficulty falling or staying asleep, even when the sleep environment is suitable, leading to reduced functioning during the day (Riemann et al. 2022). Each year, as many as 37% of the general population are believed to experience short‐term symptoms of insomnia. Chronic insomnia affects around 10% to 20% of individuals, with a higher prevalence observed among women, older adults, and those from lower socioeconomic backgrounds (Ferini‐Strambi et al. 2021). Insomnia is often triggered by stress, anxiety, depression, or poor sleeping conditions like noise or extreme temperatures (Nordin and Nordin 2016). It can also result from substance use, such as alcohol, nicotine, or illegal drugs, or shift work (Strähl 2023). It is frequently associated with increased physical and mental health issues, reduced quality of life, and greater societal burdens such as accidents, healthcare usage, and work absenteeism (Aernout et al. 2021). In recent years, the widespread impact of COVID‐19 has led to an increase in sleep‐related issues (Gupta et al. 2020). Additionally, insomnia is linked to several health conditions, including sexual dysfunction, somatic disorders, Type 2 diabetes, hypertension, schizophrenia, and heart attacks (Olfson et al. 2018).

GABA is the primary inhibitory neurotransmitter in the central nervous system (Gulias‐Cañizo et al. 2021). It functions by binding to GABA receptors (mainly GABA_A_ and GABA_B_), which reduces neuronal excitability (Benarroch 2012). This inhibitory action helps maintain a balance in brain activity and prevents overstimulation (Bassetti 2022). In anxiety and sleep disorders, there is often an imbalance in the brain's excitatory and inhibitory signals (Carbone et al. 2023). Reduced GABA activity can lead to heightened neuronal firing, contributing to symptoms such as restlessness, nervousness, and insomnia (Varinthra et al. 2024). Therefore, enhancing GABAergic transmission is a common therapeutic approach to calm the central nervous system (Gulias‐Cañizo et al. 2021). Current treatments for sleep disorders often include melatonin receptor agonists, non‐BZP hypnotics, and BZPs (McGowan et al. 2022). While these treatments are effective, prolonged use for managing insomnia may lead to side effects such as fatigue and impaired coordination, highlighting the urgent need for safer therapeutic options (Edinoff et al. 2021).

Natural products are biologically active compounds derived from natural sources, contributing significantly to drug development and disease treatment due to their therapeutic properties (Chaachouay and Zidane 2024). Various phytochemicals such as polyphenols, terpenes, alkaloids, and saponins have been associated with the sedative properties of natural sleep remedies (Zhao et al. 2014). Notably, polyphenols are well‐documented to exert their sleep‐inducing effects through interaction with GABA_A_ receptors (Cho and Shimizu 2015). A wide range of herbal and dietary supplements have traditionally been used to treat sleep disturbances and anxiety. For instance, passion flower (*Passiflora incarnata*) is used for managing anxiety and insomnia (Kaźmierczyk et al. 2024), kava kava (*Piper methysticum*) for easing tension and promoting sleep (Shinomiya et al. 2005), valerian (*Valeriana officinalis*) for improving sleep quality (Oxman et al. 2007), and Suanzaoren (*Ziziphus jujuba*) for emotional balance and better sleep (Yeh et al. 2011). These studies demonstrate that natural products have strong sedative properties and may serve as effective alternatives to synthetic sleep aids (Hosseini et al. 2024). Their interaction with neurological pathways highlights their potential in treating insomnia and anxiety (Küpeli Akkol et al. 2021).

L (+)‐tartaric acid (LTA), known by its IUPAC name (2R,3R)‐2,3‐dihydroxybutanedioic acid, is an organic compound classified as both a dicarboxylic acid and an alpha‐hydroxy acid (Tang and Yang 2018). It naturally occurs in several fruits, especially grapes (*Vitis vinifera*), where it significantly contributes to the sourness of grape juice and wine (Cholet et al. 2016). LTA has demonstrated a variety of pharmacological properties, including antihypertensive (Amssayef et al. 2023), vasorelaxant (Amssayef et al. 2024), and antiparasitic (Santos et al. 2018), as well as antihyperglycemic and lipid‐lowering effects (Amssayef and Eddouks 2022). It also shows potential neurological effects, likely linked to its influence on vascular mechanisms and receptor modulation. Studies have shown that LTA can inhibit diminazene‐induced vasorelaxation, indicating its possible role in regulating vascular dynamics that impact brain function (Kousar et al. 2022; Amssayef et al. 2023; Mohideen and Nawaz 2025). These studies suggest that LTA may possess potential relevance to neurological disorders. However, to date, no research has specifically examined its sedative properties.

Therefore, this research focused on evaluating the sedative potential of LTA using a thiopental sodium‐induced sleep model in mice, along with behavioral assessments using the open field test and rotarod performance test, alongside in silico analysis targeting the GABA_A_ receptor subunits responsible for its activity.

## 2 Materials and Methods

### 2.1 In Vivo Study

#### 2.1.1 Chemicals and Reagents

The test compound LTA (CAS No: 87‐69‐4, purity: ≥ 99.5% (GLC)) was brought from Sigma‐Aldrich (USA). Loba Chemie Pvt. Ltd. (India) provided Tween‐80 (0.5%), and Square Pharmaceuticals Ltd. (Bangladesh) supplied DZP and thiopental sodium.

#### 2.1.2 Dose Selection and Preparation

The selected doses of LTA (80 and 240 mg/kg) were based on previously published in vivo studies reporting significant neurological and vascular effects within this dose range (Amssayef et al. 2023), without any observable signs of toxicity. These doses were chosen to ensure pharmacological relevance while maintaining a favorable safety profile. A parent solution was prepared by adding a tiny amount of Tween 80 (0.5%) and adequate distilled water (DW) to obtain a concentration of 240 mg/kg. Finally, the parent solution was diluted to obtain a concentration equivalent to 80 mg/kg. In contrast, DZP as a standard drug was prepared at the dose of 2 mg/kg by adding enough DW.

#### 2.1.3 Experimental Animals

Adult *Swiss* albino mice (*Mus musculus*; b.w. 22–28 g) were purchased from the livestock section of Jahangirnagar University, Dhaka, Bangladesh. These animals were properly maintained at a constant temperature (24°C ± 3°C) (relative humidity: 65%) with controlled lighting (12‐h dark/light cycle) in the pharmacological lab of Gopalganj Science and Technology University (GSTU). In this time, the animals were provided with unlimited access to standard feeds and water ad libitum. Mice were fasted for approximately 12 h before the tests. The current investigation took place between 9:00 A.M.–3:00 P.M., and the mice were observed for 17 h to assess their potential mortality. All experimental protocols involving animals were approved by the Ethics Committee of the Department of Pharmacy, GSTU (Approval No. #gstu‐20PHR035‐01) and were performed in compliance with the ARRIVE guidelines, the NRC Guide for the Care and Use of Laboratory Animals, and OECD‐GLP recommendations.

#### 2.1.4 Study Design

In this thiopental sodium experiment, 25 mice were randomly assigned to five groups, with each group consisting of five animals (*n* = 5). The treatment groups and their specific details are presented in Table 1.

**TABLE 1:** Different treatments and their doses in sedative test.

Treatment group	Description	Dose (mg/kg)	Target receptor
Control (vehicle)	Distilled water containing 0.9% NaCl and 0.5% Tween 80	10 mL/kg	—
DZP‐2	Standard: Diazepam	2 mg/kg	GABA_A_
LTA‐80	Lower dose	80 mg/kg	Under investigation
LTA‐240	Higher dose	240 mg/kg	
DZP‐2 + LTA‐240	Test + standard combination	2 mg/kg + 240 mg/kg	
Control (vehicle): distilled water containing 0.9% NaCl and 0.5% Tween 80; DZP‐2: diazepam (dose: 2 mg/kg); LTA: L‐ (+) tartaric acid; (dose: 80 and 240 mg/kg); all treatments provided through (i.p.) route; (*n* = 5)			

Additionally, for the evaluation of locomotor activity, exploratory behavior, and motor coordination, thirty Swiss albino mice were randomly allocated into five experimental groups, with six animals in each group (*n* = 6). The treatment groups and dosing regimens were identical to those used in the thiopental‐induced sleeping test. Thirty minutes after intraperitoneal administration of the respective treatments, mice were subjected to the Open Field Test and Rotarod Test.

#### 2.1.5 Thiopental‐Induced Sleeping Test

Methodological procedures were conducted in accordance with the previously reported methodology (Hassan et al. 2024). Animals were randomly allocated to experimental groups using a simple randomization approach to minimize selection bias. All behavioral assessments and data recording were performed by investigators blinded to treatment allocation to reduce observer bias. Exclusion criteria were predefined in accordance with the principles of replacement, reduction, and refinement (3Rs) and ARRIVE guidelines. Animals exhibiting abnormal baseline behavior, signs of illness or injury prior to dosing, injection‐related complications, or failure to exhibit thiopental‐induced loss of righting reflex were excluded from analysis. No animals were excluded based on *post‐hoc* outcomes. The minimum number of animals necessary to achieve statistical validity was used, and all procedures were conducted to minimize stress, discomfort, and unnecessary use of animals. The test sample LTA (80 and 240 mg/kg), the standard drug DZP (2 mg/kg), and the control (vehicle): 10 mL/kg were administered intraperitoneally (i.p.). After 30 min, each animal was given thiopental sodium (40 mg/kg, i.p.) to induce sleep before being placed in the observation chamber. After administration of thiopental sodium, the time until loss of righting reflex and duration of sleeping time were measured up to 4 h. These percentages were calculated relative to the vehicle‐treated control group as follows:

$$\%\;\text{Incidence of sleep}=\left(\frac{\mathrm{Number}\;\mathrm{of}\;\mathrm{mice}\;\mathrm{that}\;\mathrm{slept}\;}{\mathrm{Total}\;\mathrm{mice}\;\mathrm{in}\;\mathrm{the}\;\mathrm{group}}\right)\times 100$$

$$\%\;\text{Reduction in latency}=\left(\frac{M-N}{M}\right)\times 100$$

$$\%\;\text{Increase in sleep duration time}=\left(\frac{P-Q}{Q}\right)\times 100$$

Where: *M* = mean latency of the control group, *N* = mean latency of the test or standard group, *P* = mean sleep duration in the test or standard group, *Q* = mean sleep duration in the control group.

#### 2.1.6 Open Field Test

The open‐field test was performed to assess spontaneous locomotor and exploratory activities of mice, which are commonly used indicators of central nervous system (CNS) depressant effects (Sultana et al. 2018). The apparatus consisted of a wooden arena (30 × 30 × 30 cm) with the floor divided into equal squares. Thirty minutes after treatment administration, each mouse was individually placed at the center of the apparatus and observed for 3 min.

During the observation period, the number of squares crossed (NSC) was recorded as an index of horizontal locomotor activity. The number of rearings (NR) was recorded to evaluate vertical exploratory behavior, while the number of grooming episodes (NG) was used as an indicator of self‐directed activity. Following each trial, the apparatus was thoroughly cleaned with 70% ethanol to eliminate olfactory cues.

#### 2.1.7 Rotarod Test

Following completion of the Open Field Test, motor coordination and neuromuscular performance were evaluated using a rotarod apparatus. The test was conducted to distinguish sedative‐like effects from nonspecific motor impairment or muscle relaxation (Veena et al. 2015). The rotating rod was maintained at a constant speed of 15 rpm. Thirty minutes after treatment administration, each mouse was first subjected to the Open Field Test and was subsequently assessed using the rotarod apparatus for a maximum duration of 3 min.

The latency to the first fall (s) and the total number of falls were recorded. Each animal was allowed a maximum of three falls during the testing session. The average latency to fall was calculated from all recorded trials for each mouse. A reduction in latency to fall and/or an increase in the number of falls compared with the vehicle‐treated control group was considered indicative of impaired motor coordination or reduced neuromuscular performance. DZP served as the positive control to validate the sensitivity of the assay.

#### 2.1.8 Statistical Analysis

All data, including sedative like effectiveness, are presented as mean ± standard deviation (SD). Statistical analyses were performed using GraphPad Prism (version 6.0), with comparisons among multiple groups conducted by one‐way analysis of variance (ANOVA) followed by Tukey's *post hoc* test, where appropriate. A confidence level of 95% was applied, and *P*‐values below 0.05 were considered statistically significant.

### 2.2 In Silico Study

#### 2.2.1 GABA Macromolecule Selection and Preparation

The GABA_A_ receptor was selected as the molecular target based on its well‐established involvement in sedative pharmacology. Molecular docking was performed using the experimentally resolved structure of the human GABA_A_ receptor containing the α1 and β2 subunits (PDB ID: 6 × 3X), which are known to contribute to BZP and barbiturate sensitive inhibitory neurotransmission (Bhuia et al. 2024). The receptor structure was retrieved from the RCSB Protein Data Bank (https://www.rcsb.org/, retrieved on 19 June, 2025) and prepared using PyMOL (v2.4.1) by removing crystallographic water molecules, non‐relevant chains, and heteroatoms to minimize docking artifacts. Energy minimization was carried out using the GROMOS96 force field in Swiss‐PDB Viewer to optimize receptor geometry prior to docking.

#### 2.2.2 Ligand Preparation

To get docking results, the ligand's 3D structures were downloaded from the PubChem online database (https://pubchem.ncbi.nlm.nih.gov/). These contained LTA (PubChem ID: 444305) and one standard drug, DZP (PubChem ID: 3016). The SDF format of the structures was downloaded. We modified the ligands by minimizing the energy with the MM2 (Allinger's force field) method using the Chem3D software. Given the acidic nature of LTA, ligand preparation considered its predominant ionization state under physiological pH conditions. Protonation was assigned accordingly to reflect biologically relevant charge distribution during docking, while acknowledging that dynamic pH‐dependent ionization was not explicitly modeled. Figure 1 depicts the two‐dimensional (2D) structure of LTA and DZP.

**FIGURE 1:**
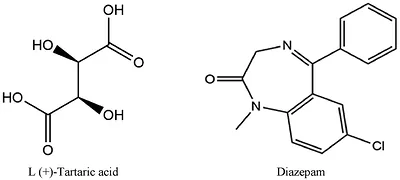
The two‐dimensional chemical structures of L (+)‐tartaric acid and diazepam.

#### 2.2.3 Docking Protocol and Non‐Bond Interactions

We utilized the PyRx computational tool to determine the binding energy of identified ligands against a target receptor. During the docking process, the target molecule was first converted into a macromolecule before importing the ligand. Subsequently, the ligand‐receptor complex was collected in PDB format and converted to PDBQT format. A grid box with dimensions 35.02 × 39.37 × 25.00 was positioned along the x, y, and z axes, and the docking computation was performed with 200 steps. The results of the docking process were saved in CSV format. The interactions between the ligand and protein were visualized using Discovery Studio (v21.1.020298) and PyMol (v2.4.1) software. Additionally, we calculated the number of HBs, identified the involved amino acid (AA) residues, and measured the bond distances.

#### 2.2.4 Pharmacokinetics, Drug‐Likeness Properties and Toxicity

In thisstudy, we used the online program SwissADME to predict the pharmacokinetics, drug‐likeness, and physiological features. Additionally, we used pkCSM, another online application, to assess the test drug's absorption, distribution, metabolism, and excretion (ADME) characteristics. Additionally, we studied the toxicity of chemical compounds and traditional pharmaceuticals using online web servers to assess a drug or substance's safety by evaluating various toxicity endpoints such as hepatotoxicity, neurotoxicity, immunotoxicity, mutagenicity, and carcinogenicity, LD_50_ values, and toxicity classes. To assess the toxicity of the molecule, we got its canonical SMILES from https://pubchem.ncbi.nlm.nih.gov/ (accessed on 19 June, 2025) and imported it into the parameter for the analysis search box of ProTox‐3.0.

## 3 Results

### 3.1 In Vivo Findings

#### 3.1.1 Thiopental Sleeping Test

The thiopental sodium‐induced sleeping test revealed significant modulation of sleep parameters across all treatment groups. One‐way ANOVA demonstrated a significant treatment effect on both sleep latency (F (4, 20) = 614.2, *p* < 0.0001, *R* ^2^ = 0.9919) and sleep duration (F (4, 20) = 531.4, *p* < 0.0001, *R* ^2^ = 0.9907).

The control group exhibited the longest latency to sleep (132.00 ± 2.54 sec). DZP‐2 significantly reduced sleep latency to 60.00 ± 2.44 sec (mean difference = 72.00 s, 95% CI: 65.32 to 78.68, adjusted *p* < 0.0001). LTA‐80 and LTA‐240 further reduced latency to 48.80 ± 3.89 sec (mean difference = 83.20 s, 95% CI: 76.52 to 89.88, adjusted *p* < 0.0001) and 44.00 ± 4.35 sec (mean difference = 88.00 s, 95% CI: 81.32 to 94.68, adjusted *p* < 0.0001), respectively. The combination group (DZP‐2 + LTA‐240) showed the shortest latency (35.00 ± 3.93 sec; mean difference = 97.00 s, 95% CI: 90.32 to 103.70, adjusted *p* < 0.0001). No significant difference was observed between LTA‐80 and LTA‐240 (mean difference = 4.80 s, 95% CI: −1.88 to 11.48, *p* = 0.2380).

The control group showed the shortest sleep duration (59.20 ± 2.38 min). DZP‐2 significantly increased sleep duration to 177.00 ± 6.51 min (mean difference = −117.80 min, 95% CI: −131.40 to −104.20, adjusted *p* < 0.0001). LTA‐80 and LTA‐240 produced substantially longer durations of 221.23 ± 8.17 min (mean difference = −162.00 min, 95% CI: −175.60 to −148.50, adjusted *p* < 0.0001) and 226.85 ± 11.87 min (mean difference = −167.60 min, 95% CI: −181.20 to −154.10, adjusted *p* < 0.0001), respectively. The combination group exhibited the longest sleep duration (238.70 ± 0.86 min; mean difference = −179.50 min, 95% CI: −193.10 to −165.90, adjusted *p* < 0.0001). No significant difference was observed between LTA‐80 and LTA‐240 (mean difference = −5.61 min, 95% CI: −19.18 to 7.95, *p* = 0.7299). Sleep incidence remained 100% across all groups. Sleep incidence remained 100% across all groups, indicating consistent induction of sleep. However, all findings related to latency and sleep duration are summarized in Figure 2.

**FIGURE 2:**
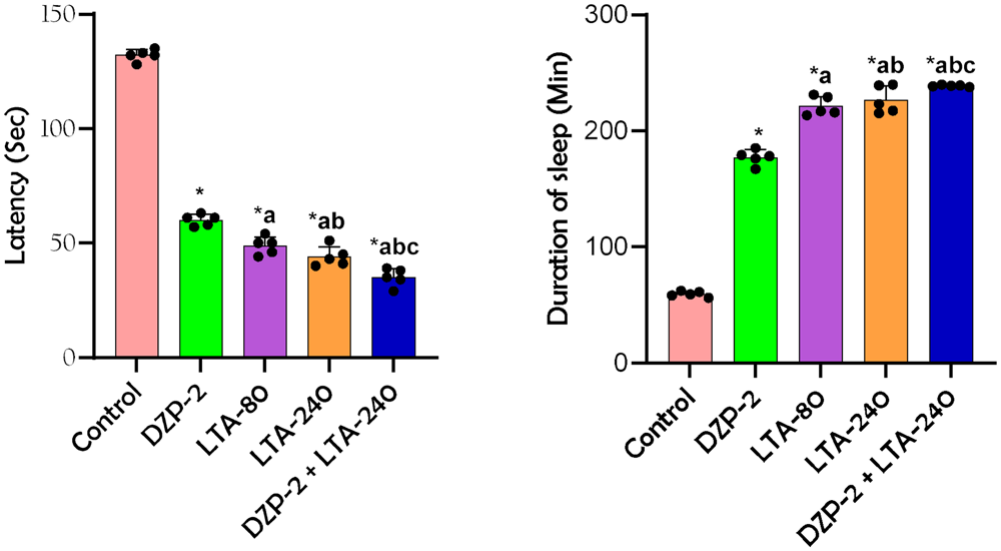
Latency and duration of sleep in different treatment groups of animals. [Values are mean ± SD (*n* = 5); One‐way ANOVA followed by *Tukey* post hoc multiple comparison test, *p <* 0.05 compared to the ^*^Control (vehicle): Distilled water containing 0.9% NaCl and 0.5% Tween 80; ^a^DZP‐2: Diazepam 2 mg/kg; ^b^LTA‐80: L (+)‐tartaric acid 80 mg/kg; ^c^LTA‐240: L (+)‐tartaric acid 240 mg/kg; ^d^DZP‐2 + LTA‐240: Diazepam 2 mg/kg + L (+)‐tartaric acid 240 mg/kg].

In vivo results again showed that all treatments significantly modulated sleep parameters compared to the control. DZP (2 mg/kg) reduced sleep latency by 54.75% and increased sleep duration by 198.98% compared to the control. LTA at 80 mg/kg showed greater effects, with a 63.19% decrease in latency and a 273.69% increase in sleep duration. At 240 mg/kg, LTA also significantly improved both parameters with a 66.81% decrease in latency and a 283.19% increase in sleep duration. The combination of LTA (240 mg/kg) with DZP had the most pronounced effect, reducing latency by 73.60% and increasing sleep duration by 303.20%. This suggests an enhanced sedative like effect when both agents are used together. However, all the data about latency decrease and sleeping time increase are given in Table 2.

**TABLE 2:** Percentage modulation of latency and duration of sleep by the test and/or standard groups compared to the vehicle (control) group.

Treatments	Latency decrease (%)	Sleeping time increase (%)
Control (Vehicle)	—	—
DZP‐2	54.75	198.98
LTA‐80	63.19	273.69
LTA‐240	66.81	283.19
DZP‐2 + LTA‐240	73.60	303.20

*Note*: Control (vehicle): distilled water containing 0.9% NaCl and 0.5% Tween 80; DZP‐2: diazepam 2 mg/kg; LTA‐80: L (+)‐tartaric acid 80 mg/kg; LTA‐240: L (+)‐tartaric acid 240 mg/kg; DZP‐2 + LTA‐240: diazepam 2 mg/kg + L (+)‐tartaric acid 240 mg/kg.

#### 3.1.2 Effect on General Locomotor and Exploratory Activity

The number of square crossings was significantly reduced following treatment, with a significant overall group effect (F (4, 25) = 253.952, *p* = 7.55 × 10^−20^, 95% CI: 0.959–1.000). Significant differences were observed between DZP‐2 and control (mean difference = −49.5, 95% CI: −55.5442 to −43.4558, *p* = 3.28 × 10^−14^), LTA‐80 and control (−21.0, 95% CI: −27.0442 to −14.9558, *p* = 2.05 × 10^−9^), LTA‐240 and control (−42.5, 95% CI: −48.5442 to −36.4558, *p* = 3.30 × 10^−14^), and DZP‐2 + LTA‐240 and control (−56.6667, 95% CI: −62.7109 to −50.6224, *p* = 3.28 × 10^−14^). For grooming, the groups also differed significantly (F (4, 25) = 8.03, *p* = 0.000263, 95% CI: 0.282–1.000). DZP‐2 and DZP‐2 + LTA‐240 differed significantly from control (mean differences = −2.8333 and −2.50, 95% CI: −4.5346 to −1.1321 and −4.2013 to −0.7988, *p* = 0.000438 and 0.00188, respectively), whereas LTA‐80 and LTA‐240 did not (*p* = 0.437 and 0.289, respectively). Similarly, rearing differed significantly among groups (F (4, 25) = 27.29, *p* = 8.45 × 10^−9^, 95% CI: 0.679–1.000), with significant differences between control and DZP‐2 (mean difference = −14.8333, 95% CI: −19.4146 to −10.2521, *p* = 8.42 × 10^−9^), LTA‐80 (−10.1667, 95% CI: −14.7479 to −5.5854, *p* = 7.40 × 10^−6^), LTA‐240 (−10.8333, 95% CI: −15.4146 to −6.2521, *p* = 2.63 × 10^−6^), and DZP‐2 + LTA‐240 (−13.0, 95% CI: −17.5813 to −8.4187, *p* = 1.05 × 10^−7^).

The open‐field test demonstrated that LTA treatment reduced spontaneous locomotor and exploratory activities in a dose‐dependent manner (Figure 3). The vehicle‐treated control group exhibited the highest number of square crossings (71.33 ± 2.34), rearings (25.00 ± 4.05), and grooming episodes (3.00 ± 1.26). DZP markedly decreased all behavioral parameters. Similarly, LTA‐80 and LTA‐240 reduced locomotor activity, exploratory behavior, and grooming frequency compared with the control group, with the higher dose producing a more pronounced effect. The combination of LTA‐240 and DZP produced the greatest suppression of locomotor and exploratory activities.

**FIGURE 3:**
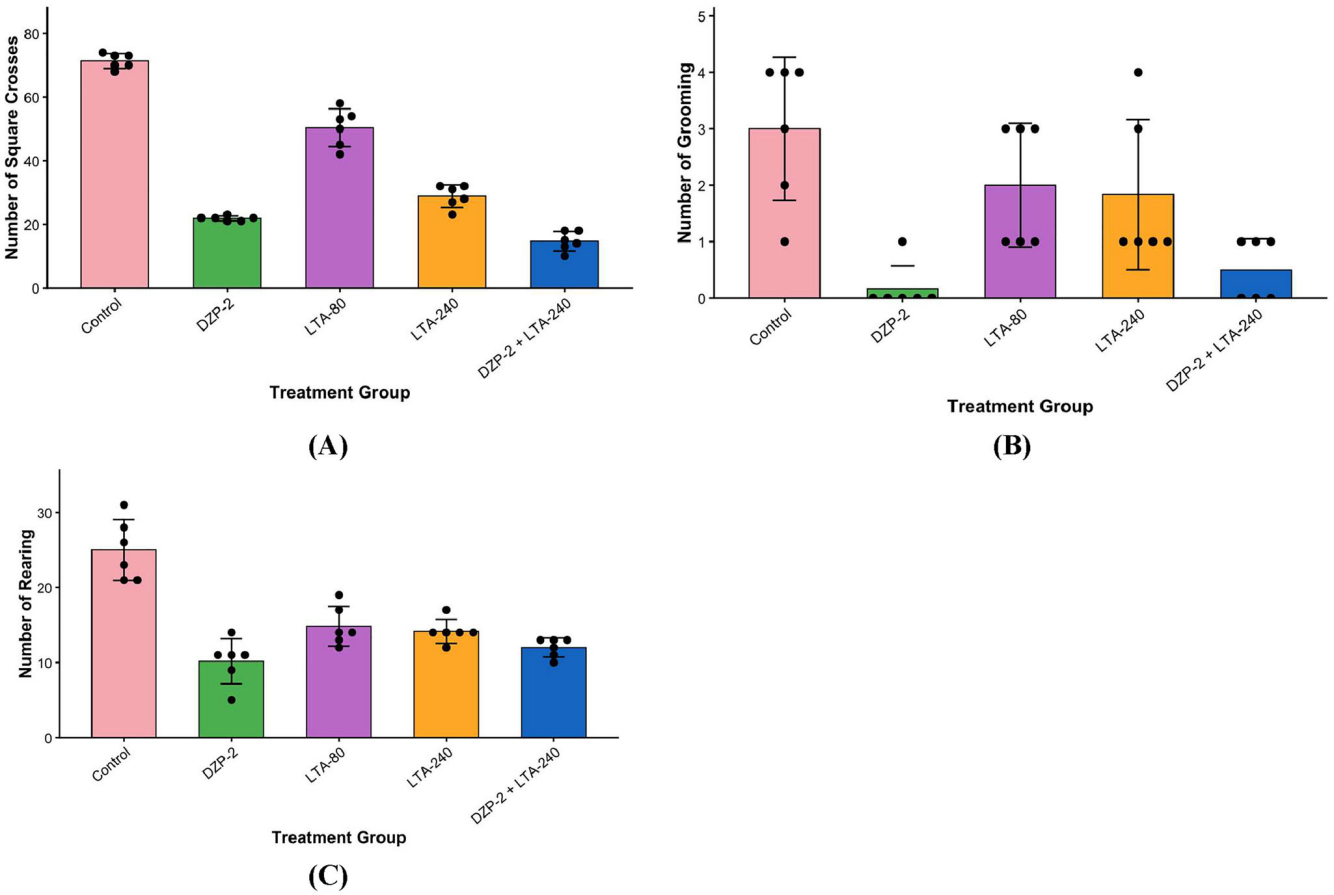
Effects of LTA on locomotor activity in the open‐field test. **(A)** Number of squares crossed (NSC), **(B)** Number of grooming episodes (NG), and **(C)** Number of rearings (NR). [Values are mean ± SD (*n* = 6); One‐way ANOVA followed by Tukey post hoc multiple comparison test, *p* < 0.05 compared to the Control (vehicle): Distilled water containing 0.9% NaCl and 0.5% Tween 80; DZP‐2: Diazepam 2 mg/kg; LTA‐80: L (+)‐tartaric acid 80 mg/kg; LTA‐240: L (+)‐tartaric acid 240 mg/kg; DZP‐2 + LTA‐240: diazepam 2 mg/kg + L (+)‐tartaric acid 240 mg/kg. All treatments were administered via the intraperitoneal (i.p.) route.].

#### 3.1.3 Effect on Motor Coordination

In the rotarod test, the latency to fall was significantly affected by treatment (F (4, 25) = 408.798, *p* = 2.22 × 10^−22^, 95% CI: 0.974–1.000). Pairwise analysis showed significant differences between DZP‐2 and NC (mean difference = −47.6944 s, 95% CI: −51.8978 to −43.4911, *p* = 3.28 × 10^−14^), LTA‐80 and NC (−26.4167 s, 95% CI: −30.62 to −22.2133, *p* = 3.74 × 10^−14^), LTA‐240 and NC (−43.3611 s, 95% CI: −47.5645 to −39.1577, *p* = 3.28 × 10^−14^), and DZP‐2 + LTA‐240 and NC (−47.8056 s, 95% CI: −52.0089 to −43.6022, *p* = 3.28 × 10^−14^). The DZP‐2 + LTA‐240 group did not differ significantly from DZP‐2 (−0.1111 s, 95% CI: −4.3145–4.0923, *p* > 0.9999).

Rotarod testing revealed a dose‐dependent reduction in motor coordination following LTA administration (Table 3 and Figure 4). Control animals remained on the rotating rod for the longest duration (55.00 ± 4.48 s), whereas diazepam‐treated mice showed a marked decrease in performance (7.94 ± 2.14 s). LTA treatment reduced latency to fall at both tested doses, with LTA‐240 showing a greater effect than LTA‐80. The combination of LTA‐240 and DZP resulted in the lowest rotarod performance. These findings indicate that LTA exerts CNS depressant effects accompanied by moderate impairment of motor coordination, although the impairment remained less pronounced than that produced by DZP at the lower dose.

**TABLE 3:** Effect on motor coordination by the test and/or standard groups compared to the vehicle (control) group.

Group	Latency to first fall (s)	Number of falls	Average latency (s)
NC	51.33 ± 5.99	2.50 ± 0.54	55.00 ± 4.48
DZP‐2	5.50 ± 2.43	3.00 ± 0.00	7.94 ± 2.14
LTA‐80	38.50 ± 6.72	3.00 ± 0.00	29.22 ± 13.16
LTA‐240	11.00 ± 2.28	3.00 ± 0.00	12.28 ± 1.11
DZP‐2 + LTA‐240	5.67 ± 1.53	3.00 ± 0.00	7.83 ± 1.91

*Note*: Values are mean ± SD (standard deviation); One‐way ANOVA followed by *Tukey* multiple comparison test, *p <* 0.05 compared to the NC: Control (vehicle), distilled water containing 0.9% NaCl and 0.5% Tween 80; DZP‐2: diazepam 2 mg/kg; LTA‐80: L (+)‐tartaric acid 80 mg/kg; ^c^LTA‐240: L (+)‐tartaric acid 240 mg/kg; DZP‐2 + LTA‐240: diazepam 2 mg/kg + L (+)‐tartaric acid 240 mg/kg; All treatments provided through (i.p.) route; (*n* = 6).

**FIGURE 4:**
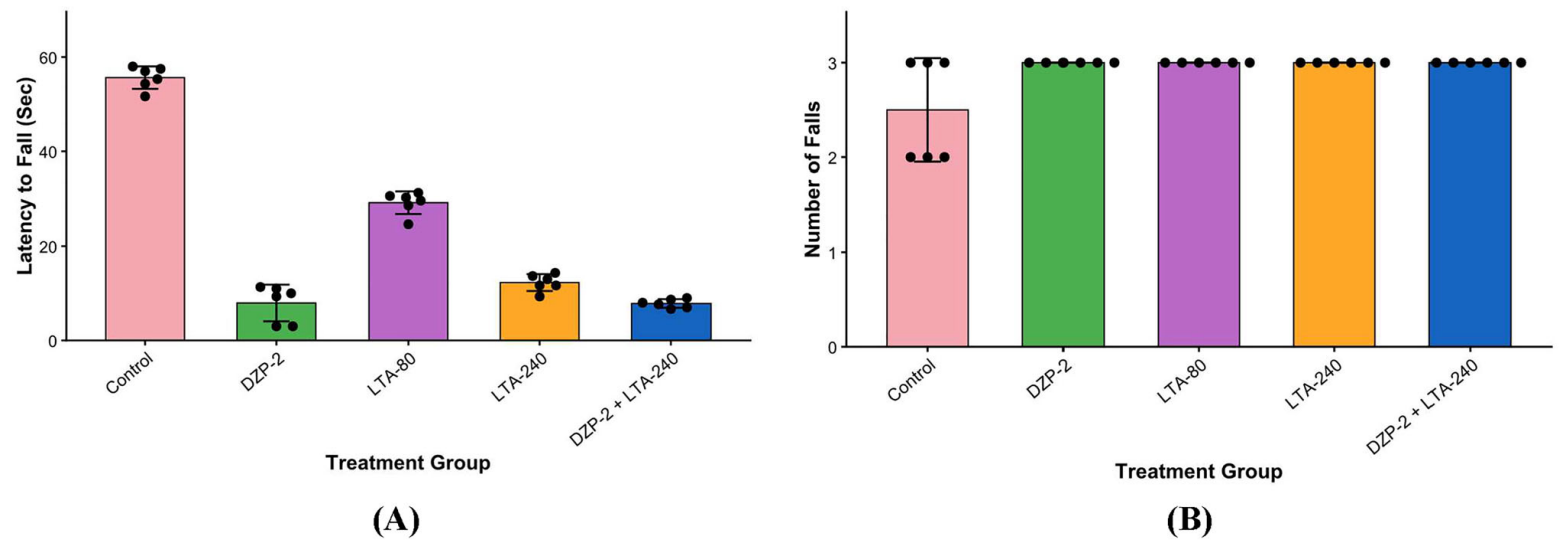
Effects of LTA on motor coordination in the rotarod test. (A) Latency to first fall (s) and (B) number of falls. [Values are mean ± SD (*n* = 6); one‐way ANOVA followed by Tukey post hoc multiple comparison test, *p* < 0.05 compared to the control (vehicle): distilled water containing 0.9% NaCl and 0.5% Tween 80; DZP‐2: diazepam 2 mg/kg; LTA‐80: L (+)‐tartaric acid 80 mg/kg; LTA‐240: L (+)‐tartaric acid 240 mg/kg; DZP‐2 + LTA‐240: diazepam 2 mg/kg + L (+)‐tartaric acid 240 mg/kg. All treatments were administered via the intraperitoneal (i.p.) route. Latency to first fall (s) = time elapsed before the first fall from the rotating rod; number of falls = total falls recorded during the test period; average latency (s) = mean time maintained on the rotating rod.]

### 3.2 In Silico Findings

#### 3.2.1 Molecular Docking and Visualization of Ligand‐Receptor Interaction

The in silico analysis showed that DZP had a stronger binding affinity (–8.3 kcal/mol) for GABA_A_ receptors compared to LTA, which showed a lower score (–4.8 kcal/mol). As shown in Figure 5B, LTA established two HBs with the GABA_A_ receptor at ASN A:265 (2.67 Å) and THR A:266 (3.48 Å). In contrast, DZP formed a single HB with LEU A: 285 (3.54 Å) and exhibited several hydrophobic (HP) interactions with residues such as PHE A: 289, PRO B: 233, MET B: 236, MET A: 286, and LEU B: 232 (Figure 5A). Table 4 presents the docking scores, number and lengths of HBs, along with the AA residues involved in interactions with the ligands. Figures 5A,B illustrate the 2D and 3D representations of non‐bonded interactions between DZP and LTA with the GABA_A_ receptor subunits α1 and β2 (PDB ID: 6 × 3X).

**FIGURE 5:**
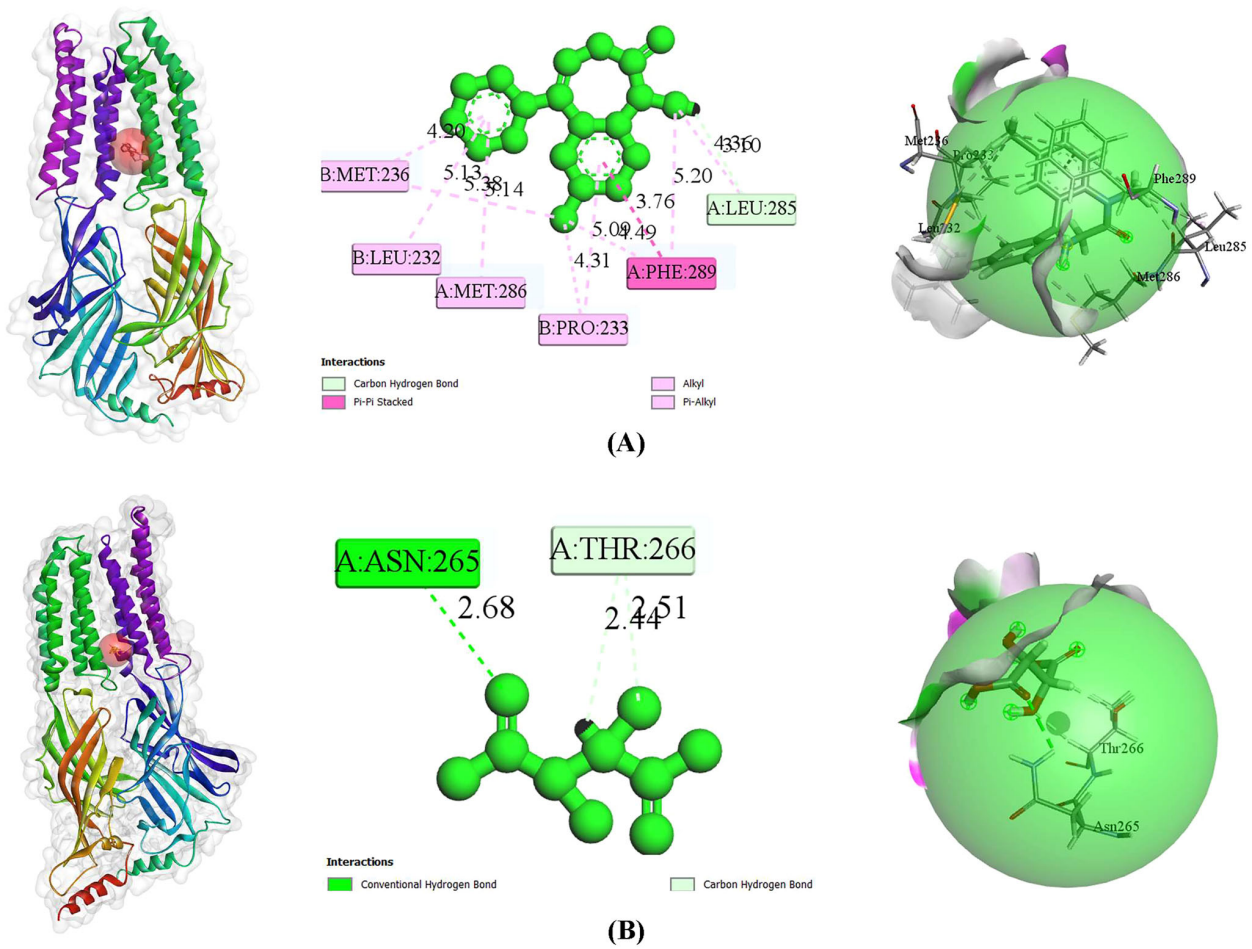
Two‐dimensional (2D) and three‐dimensional (3D) non‐bond interactions of diazepam and L (+)‐tartaric acid with the Gamma‐aminobutyric acid A (GABA_A_)​ receptor (α1 and β2 subunits; PDB ID: 6×3X). **5 (A)**: 2D and 3D interactions of diazepam with the GABA_A​_ receptor. 5 **(B)**: 2D and 3D interactions of L (+)‐tartaric acid with the GABA_A_​ receptor.

**TABLE 4:** Molecular docking results of diazepam and L (+)‐tartaric acid towards the GABAA receptor (α1 and β2 subunits).

Ligands	Receptors (PDB ID)	BA (kcal/mol)	No of HB	Amino acid residues	
				HBs	Others
DZP	GABA_A_ receptors (α1 and β2 subunits) (6 × 3X)	−8.3	1	LEU A: 285 (3.54)	PHE A: 289 PRO B: 233 MET B: 236 MET A: 286 LEU B: 232
LTA		−4.8	2	ASN A: 265 (2.67) THR A: 266 (3.48)	—

Abbreviations: AA: amino acid; BA: binding affinity; DZP: diazepam; HB: hydrogen bond; LTA: L (+)‐tartaric acid; PDB ID: protein data bank identification code.

#### 3.2.2 Estimation of Drug‐Likeness and Pharmacokinetics

LTA exhibits favorable physicochemical properties with a molecular weight of 150.09 g/mol and high water solubility (log S = 0.61). It complies with Lipinski's rule, indicating good drug‐likeness and moderate bioavailability (score 0.56). However, it shows poor intestinal absorption (3.709%) and low Caco‐2 permeability. It does not inhibit or act as a substrate for P‐glycoprotein. It is not a substrate or inhibitor of major CYP enzymes, indicating low metabolic interaction. Total clearance is moderate (0.885 log ml/min/kg), and it is not a renal OCT2 substrate. However, all the parameters related to this criterion are presented in Table 5, and its physiochemical properties are shown in Figure 6.

**TABLE 5:** Pharmacokinetic properties of L (+)‐tartaric acid predicted by SwissADME and pkCSM online server.

Properties	Parameters	L (+)‐tartaric acid
Physicochemical properties	Formula	C_4_H_6_O_6_
	MW (g/mol)	150.09
	No. of heavy atoms	10
	No. of aromatic heavy atoms	0
	No. of HBD	4
	No. of HBA	6
	MR	27.21
Lipophilicity	Log *P*_o/w_ (XLOGP3)	−1.88
Drug‐likeness	Lipinski	Yes; 0 violation
	Bioavailability score	0.56
Water solubility	Log S (ESOL)	0.61
	Class	Highly soluble
Absorption	Caco‐2 permeability (log Papp in 10^−6^ cm/s)	−0.374
	Intestinal absorption (human) numeric (% Absorbed)	3.709
	Skin permeability (log Kp cm/h)	−2.735
	P‐glycoprotein I inhibitor	No
	P‐glycoprotein II inhibitor	No
Distribution	BBB permeability (log BB)	−0.867
	CNS permeability (log PS)	−3.987
	VDss (human) (log L/kg)	−0.847
Metabolism	CYP2D6 substrate	No
	CYP3A4 substrate	No
	CYP2D6 inhibitor	No
	CYP3A4 inhibitor	No
Excretion	Total clearance (log ml/min/kg)	0.885
	Renal OCT2 substrate	No

Abbreviations: BBB: blood‐brain barrier; CNS: central nervous system; CYP: cytochrome; HBA: hydrogen bond acceptors; HBD: hydrogen bond donors; MR: molar refractivity; MW: molecular weight; P450; VDss: volume of distribution (at steady state).

**FIGURE 6:**
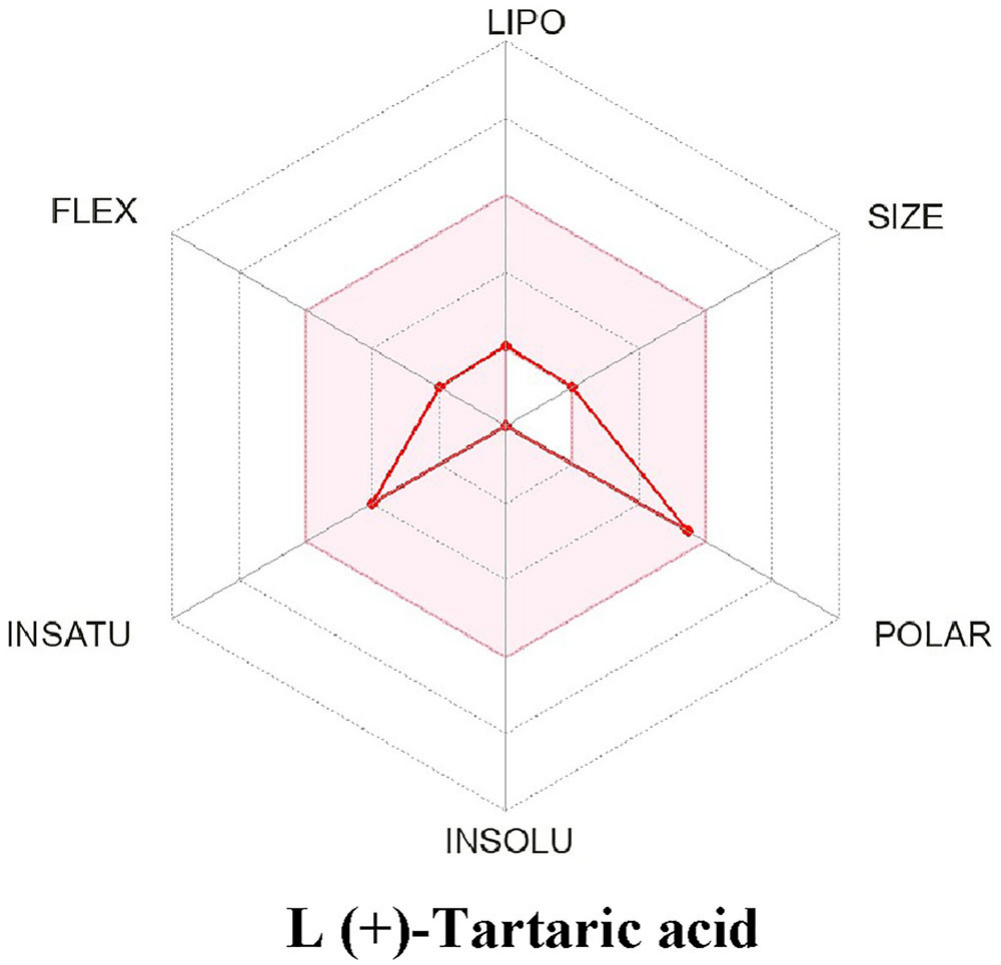
Summarizes the L (+)‐tartaric acid physicochemical properties. [The physicochemical space that is appropriate for oral bioavailability is the colored zone; SIZE: 150 g/mol < MV < 500 g/mol; LIPO (lipophilicity): –7 < XLOGP3 < + 5.0; INSOLU (insolubility): –6 < log S (ESOL) < 0; POLAR (polarity): 20 Å^2^ < TPSA < 130 Å^2^; IN‐SATU (in saturation): 0.25 < fraction Csp3 < 1; FLEX (flexibility): 0 < num. rotatable bonds < 9].

#### 3.2.3 Evaluation of the Predicted Toxicity Profiles of the Selected Compound

The toxicity profile of LTA, predicted using the ProTox 3.0 server, indicates low toxicity, with a toxicity class of 5 and a predicted LD_50_ of 2497 mg/kg. It is considered inactive for hepatotoxicity, neurotoxicity, immunotoxicity, mutagenicity, and carcinogenicity, suggesting a favorable safety profile with minimal toxicological risks (Table 6).

**TABLE 6:** The toxicity profile of L (+)‐tartaric acid with different parameters predicted by the ProTox 3.0 server.

Properties	Parameters	L (+)‐tartaric acid
Toxicity	TC	5
	Predicted LD_50_ (mg/kg)	2497
	Hepatotoxicity	Inactive
	Neurotoxicity	Inactive
	Immunotoxicity	Inactive
	Mutagenicity	Inactive
	Carcinogenicity	Inactive

*Note*: TC: Toxicity class; LD_50_: Median lethal dose.

## 4 Discussion

GABA is the main inhibitory neurotransmitter in the CNS (Gulias‐Cañizo et al. 2021). It binds to GABA_A_ receptors, allowing chloride ions to enter neurons, causing hyperpolarization (Knoflach et al. 2016). This reduces neuronal excitability and inhibits signal transmission (Knoflach et al. 2016). In sedation, increased GABA activity suppresses brain regions responsible for alertness and arousal (Harrison 2007). This leads to decreased consciousness, relaxation, and reduced responsiveness. Overall, the GABAergic pathway plays a key role in maintaining calm and promoting sleep (Oishi et al. 2023).

GABA_A_ receptors are pentameric ligand‐gated chloride channels that mediate rapid inhibitory neurotransmission in the CNS. GABA binds to two orthosteric sites located at the extracellular α1 and β2 subunit interfaces, whereas benzodiazepines bind to an allosteric site at the α^+^/γ^–^ interface and enhance GABA‐mediated receptor activity rather than directly activating the receptor (Olsen 2018). BZPs and barbiturates are CNS depressants that enhance the activity of GABA, the brain's main inhibitory neurotransmitter (Alkattan et al. 2021). BZPs bind to specific sites on the GABA_A_ receptor to increase its calming effects, while barbiturates act at a different site to prolong inhibition (Philip et al. 2025). Both are used for their sedative and anxiolytic properties, though barbiturates carry a higher risk of dependence and overdose. Deletion of α1 or β2 subunits reduces sedative/hypnotic drug effects, with α1 mediating zolpidem action and β2 specifically required for etomidate‐induced sedation (Blednov et al. 2003). Furthermore, β2 subunits mediate sedation, whereas β3 subunits are sufficient for anesthesia, revealing a functional separation between these isoforms (Reynolds et al. 2003). However, DZP is used as a positive allosteric modulator of GABA_A_ receptor in sedative tests because it binds to the BZP site on the GABA_A_ receptor, enhancing GABA's inhibitory action and increasing sedation (Brohan and Goudra 2017). This helps in evaluating sedative activity by promoting sleep or reducing anxiety‐like behavior (Pádua‐Reis et al. 2021).

TS, a barbiturate, works as a sedative inducer by enhancing the activity of GABA at the GABA_A_ receptor (Mednikova et al. 2019). It increases the duration of chloride ion channel opening, leading to prolonged hyperpolarization of neurons and stronger CNS depression (Liu and Song 2025). This results in rapid onset of sleep, making it effective for inducing sedation in experimental models. Its action helps assess the sedative potential of test substances by measuring sleep onset and duration (Mednikova et al. 2019). However, this model primarily reflects potentiation of barbiturate‐induced hypnosis and does not fully characterize intrinsic sedative activity or distinguish sedation from motor impairment; therefore, the observed sedative‐like effect of LTA should be interpreted within this context (Mihic and Harris 2011).

In vivo findings clearly indicate that both LTA‐80 and LTA‐240 produced significantly greater sedative‐like effects than the control and DZP‐2 groups, as evidenced by the marked reduction in sleep latency and substantial increase in sleep duration. Notably, LTA‐240 showed a slightly higher effect than LTA‐80, indicating a dose‐dependent sedative‐like effect. Furthermore, the combination of DZP‐2 and LTA‐240 resulted in the most pronounced sedative‐like effect, with the shortest latency (35.00 ± 3.93 sec) and the longest sleep duration (238.70 ± 0.86 min), suggesting an enhancing interaction. These results strongly support the potential of LTA, especially in combination therapy, as like a potent sedative agent. Within the tested dose range, LTA produced a dose‐related enhancement of thiopental‐induced hypnosis. These findings do not establish greater potency than DZP, because dose‐equivalent comparisons and full dose‐response analyses were not performed. However, this model primarily reflects potentiation of barbiturate‐induced hypnosis and does not fully characterize intrinsic sedative activity or distinguish sedation from motor impairment; therefore, the observed effects of LTA should be interpreted as sedative‐like rather than definitive intrinsic sedation. The possible sedative mechanism of LTA is shown in Figure 7. Accordingly, Figure 7 should be interpreted as a conceptual and hypothesis‐generating model rather than a representation of a confirmed mechanistic pathway.

**FIGURE 7:**
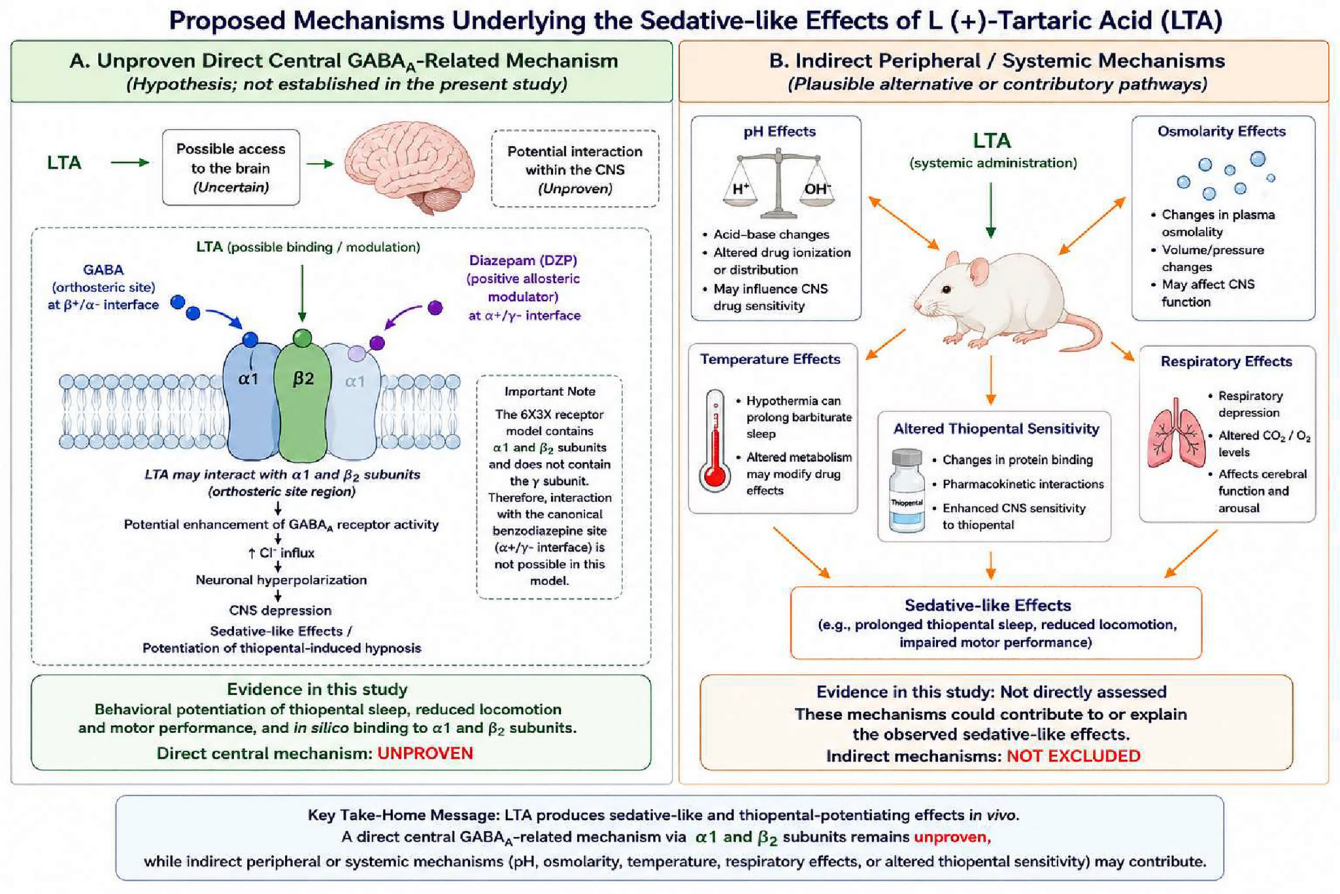
Proposed mechanisms underlying the sedative‐like effects of LTA. [Two potential pathways are illustrated: (A) a hypothesized direct central GABA_A_‐related mechanism, which remains unproven, and (B) plausible indirect peripheral or systemic mechanisms (pH, temperature, osmolarity, or respiratory effects). The present study provides behavioral evidence for LTA‐induced potentiation of thiopental sleep and reduced locomotor and motor performance, along with in silico binding data to α1 and β2 subunits. However, direct central GABA_A_ receptor involvement has not been confirmed, and indirect mechanisms have not been excluded.]

Previous in vitro work by Ha and Basile (1996) demonstrated that ammonium tartrate enhances [^3^H]flunitrazepam binding to the benzodiazepine site of the GABA_A_ receptor complex, with both GABA‐dependent and concentration‐dependent biphasic effects on ligand binding affinity (Ha and Basile 1996). This finding suggests that tartrate‐containing compounds may influence receptor binding characteristics under controlled experimental conditions. However, such in vitro binding modulation does not directly translate to functional or behavioral outcomes in vivo. Therefore, while this evidence may support a potential modulatory interaction with the GABAergic system, it should be interpreted cautiously and does not establish a confirmed mechanism underlying the sedative‐like effects observed in the present study.

To further characterize the behavioral effects of LTA and address potential confounding factors associated with the thiopental‐induced sleeping test, open‐field and rotarod assessments were conducted. In the open‐field test, LTA produced a dose‐dependent reduction in the number of square crossings, rearing frequency, and grooming behavior, indicating decreased spontaneous locomotor and exploratory activity. These findings are consistent with the behavioral profile of CNS depressant and sedative agents and support the observed potentiation of thiopental‐induced sleep. Similar reductions in locomotor activity have been reported for compounds exhibiting sedative‐like properties through modulation of GABAergic neurotransmission.

The rotarod test was performed to distinguish sedative effects from nonspecific motor impairment or muscle relaxation (Veena et al. 2015). LTA treatment resulted in a dose‐dependent decrease in latency to fall, suggesting some degree of impairment in motor coordination and neuromuscular performance. However, the reduction observed at the lower dose remained less pronounced than that produced by diazepam, a well‐established benzodiazepine sedative. These findings suggest that the behavioral effects of LTA are not solely attributable to severe motor dysfunction but rather reflect a CNS depressant profile characterized by reduced locomotor activity accompanied by moderate impairment of motor coordination. The rotarod findings indicate that motor impairment contributes to the behavioral phenotype, especially at the higher LTA dose. Therefore, the reduction in locomotor activity and the potentiation of thiopental‐induced sleep cannot be attributed exclusively to sedation.

Importantly, the combined administration of LTA and DZP produced greater suppression of locomotor activity and rotarod performance than either treatment alone, paralleling the enhancement of thiopental‐induced hypnosis observed in the sleeping test. This observation further supports the possibility that LTA may interact with pathways involved in GABAergic neurotransmission, although the precise mechanism remains to be elucidated. Taken together, the results of the open‐field, rotarod, and thiopental‐induced sleeping tests provide complementary evidence supporting the sedative‐like activity of LTA while helping to differentiate sedation from generalized behavioral suppression. The effects of LTA on locomotor activity, exploratory behavior, and motor coordination as assessed by the Open Field and rotarod tests are illustrated in Figure 8.

**FIGURE 8:**
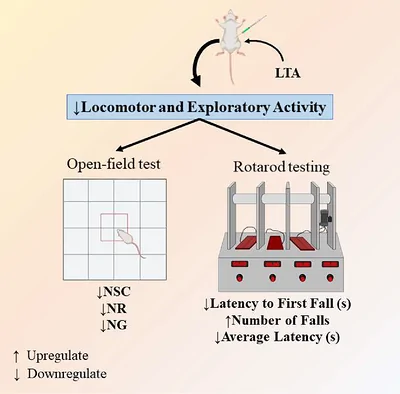
Effects of LTA on behavioral parameters. [LTA decreased locomotor and exploratory activity in the open‐field test (↓NSC, ↓NR, ↓NG) and impaired motor coordination in the rotarod test (↓latency to fall, ↑number of falls, ↓average latency). NSC: number of square crossings; NR: number of rearings; NG: number of grooming episodes.]

Despite the inclusion of these additional behavioral assessments, some limitations remain. Physiological parameters such as body temperature and respiratory rate were not monitored during the thiopental‐induced sleeping test. Because these factors can influence sensitivity to barbiturate‐induced hypnosis, future studies should incorporate physiological monitoring together with receptor‐specific antagonism, pharmacokinetic evaluation, and brain penetration studies to further clarify the mechanism underlying the sedative‐like effects of LTA.

Molecular docking is a computational method used to simulate how a ligand interacts with a target macromolecule, like a protein or nucleic acid (Feng et al. 2022). It plays a crucial role in drug discovery and structural biology by predicting binding affinity and biological activity and helping to optimize lead compounds for further research and development (Verma and Pathak 2022). *In‐silico* analysis revealed that LTA exhibited a lower binding affinity (–4.8 kcal/mol) for GABA_A_ receptors compared to DZP (–8.3 kcal/mol), with no overlapping AA residues in their interactions. However, LTA formed two HBs with key residues ASN A: 265 and THR A: 266 at favorable distances, which may contribute to receptor stabilization. However, a compound with lower binding affinity can still produce strong sedative effects because binding affinity and efficacy are not the same. A study by Kuriyama and Tomono (1986) demonstrated that 450191‐S produced strong sedative and hypnotic effects in animal models despite its very weak binding to BZP receptors. Instead, its sedative effects correlated with enhanced low‐affinity GABA_A_ receptor binding, indicating that sedation can occur through GABAergic mechanisms independent of direct BZP receptor interaction (Kuriyama and Tomono 1986). This study demonstrated that a drug exhibiting lower binding affinity for the GABA_A_ receptor can still possess significant sedative‐like potential. Additionally, it may act as a positive allosteric modulator at a different site on the GABA_A_ receptor than DZP, enhancing GABAergic activity more strongly. Such allosteric modulation can significantly boost receptor function even with weaker binding.

Pharmacokinetics prediction estimates how a drug is ADME in the body. It helps optimize dosage, ensure safety, and improve drug development (Sucharitha et al. 2022). Solubility is crucial for drug absorption and bioavailability. Poor solubility can reduce therapeutic effect and complicate formulation (Stielow et al. 2023). Lipinski's rule helps identify drug‐like compounds by predicting good oral bioavailability based on molecular properties like size, lipophilicity, and hydrogen bonding (Nhlapho et al. 2024). Pharmacokinetics prediction indicated that LTA complies with all of Lipinski's rules and shows high water solubility, suggesting acceptable drug‐likeness; however, the very low predicted intestinal absorption (∼3.7%) indicates that oral administration is currently not feasible without formulation optimization. Therefore, these findings should be considered proof‐of‐concept, and further pharmacokinetic and formulation studies are required. The poor BBB permeability (logBB −0.867; logPS −3.987) findings raise uncertainty regarding its ability to reach the CNS at pharmacologically relevant concentrations. Given the predicted poor intestinal absorption and limited BBB/CNS permeability, the translational potential of LTA remains preliminary. Oral feasibility and central target engagement require experimental pharmacokinetic confirmation. Therefore, the proposed interaction with GABA_A_ receptors should be interpreted as hypothetical rather than confirmed. The observed potentiation of thiopental‐induced sleep may alternatively be explained by indirect peripheral mechanisms or systemic effects, rather than direct central receptor modulation. Its low molecular weight and moderate bioavailability further support its potential as a drug candidate. Although its lack of interaction with P‐glycoprotein and CYP enzymes minimizes the risk of metabolic and transporter‐related issues. Overall, these properties indicate that LTA, with further formulation or delivery improvement, could be developed as a promising therapeutic agent.

Toxicity prediction involves applying computational approaches to evaluate the possible adverse effects of compounds on living organisms (Pérez Santín et al. 2021). Toxicity prediction showed that LTA shows low toxicity (LD_50_ 2497 mg/kg) and is inactive for hepatotoxicity, neurotoxicity, immunotoxicity, mutagenicity, and carcinogenicity, indicating a favorable safety profile. These results suggest minimal toxicological risk for further development.

Overall, the present study demonstrates that LTA at doses of 80 and 240 mg/kg exerts significant sedative‐like effects in vivo, with the higher dose producing a more pronounced response and further potentiating the activity of DZP. The thiopental sodium‐induced sleeping test revealed a dose‐dependent enhancement of hypnotic activity, while the open‐field test showed significant reductions in locomotor and exploratory behaviors, including decreases in square crossings, rearing, and grooming frequencies, consistent with CNS depressant activity. Furthermore, rotarod assessment demonstrated a dose‐dependent reduction in motor coordination and neuromuscular performance. However, the impairment observed with LTA remained less pronounced than that produced by DZP at the lower tested dose, suggesting that the observed behavioral effects are primarily attributable to sedation rather than severe motor dysfunction. The greater suppression observed in the LTA‐DZP combination group across all behavioral paradigms further supports a potential interaction with CNS inhibitory pathways. The behavioral findings are compatible with a CNS depressant‐like profile and may be consistent with modulation of inhibitory neurotransmission. However, direct GABA_A_ receptor involvement remains unproven and requires pharmacological antagonism, electrophysiology, receptor‐binding studies, and pharmacokinetic confirmation.

In parallel, molecular docking analysis revealed a lower binding affinity of LTA toward the GABA_A_ receptor compared with DZP; nevertheless, the formation of multiple hydrogen bonds with key amino acid residues suggests a potentially stable receptor interaction capable of contributing to receptor modulation. Collectively, the behavioral and computational findings support the hypothesis that LTA may influence GABAergic neurotransmission, possibly through positive allosteric modulation at a site distinct from the classical benzodiazepine‐binding pocket. Despite these promising findings, several limitations should be acknowledged. Physiological parameters such as body temperature and respiratory rate were not monitored during the thiopental‐induced sleeping test, and the potential contribution of physicochemical factors associated with the acidic nature of LTA cannot be completely excluded because pH‐matched controls or neutralized formulations were not evaluated. However, a major limitation of this study is the lack of pH‐ and osmolarity‐matched controls, as LTA is a dibasic acid that may alter local pH and osmolarity following intraperitoneal administration, potentially influencing physiology, stress responses, and thiopental sensitivity. Moreover, the greater sedative‐like effect observed with LTA relative to DZP should not be interpreted as evidence of greater pharmacological potency, as LTA was administered at substantially higher doses than DZP. Future studies should therefore incorporate physicochemical controls, pharmacokinetic and brain penetration analyses, receptor‐specific antagonists, electrophysiological investigations, and formal synergy assessments to clarify the precise mechanism of action, pharmacodynamic interactions, and translational potential of LTA as a candidate sedative agent.

## 5 Conclusion

In conclusion, LTA exhibits significant sedative‐like activity in vivo, as evidenced by its ability to enhance thiopental sodium‐induced hypnosis, suppress locomotor and exploratory behaviors in the open‐field test, and reduce motor coordination in the rotarod assay. These effects occurred in a dose‐dependent manner and were further potentiated when LTA was combined with DZP. Molecular docking analyses revealed favorable interactions with the GABA_A_ receptor, supporting a possible role of GABAergic modulation in the observed pharmacological effects. Although the precise mechanism remains to be established, the combined in silico and behavioral findings provide proof‐of‐concept evidence that LTA possesses CNS depressant and sedative‐like properties. Future studies incorporating receptor‐specific antagonists, electrophysiological investigations, pharmacokinetic analyses, and physicochemical controls are warranted to confirm the underlying mechanism and evaluate the translational potential of LTA as a candidate sedative agent.

## Author Contributions

**Sumaya Akter Bithi**: conceptualization, Writing – original draft, Writing – review and editing, formal analysis, methodology, investigation, visualization, data curation. **Anike Chakrabarty**: visualization, software, resources, writing – original draft, writing – review and editing. **Khadija Akter**: conceptualization, writing – original draft, writing – review and editing, formal analysis, project administration, supervision, data curation. **Chinmoy Kumar Saha**: writing – original draft, visualization, software, resources. **Mohammad Y. Alshahrani**: conceptualization, writing – original draft, writing – review and editing, methodology, visualization, formal analysis, resources. **Md. Arif Hossain**: investigation, validation, methodology. **Emon Mia**: investigation, validation, visualization, writing – original draft, writing – review and editing. **Noshin Tasnim Yana**: investigation, software, resources, visualization. **Afiya Ajmee**: visualization, software, resources. **Proma Mandal**: visualization, software, resources, formal analysis. **Md. Sakib Al Hasan**: conceptualization, writing – original draft, writing – review and editing, formal analysis, project administration, supervision. **Muhammad Torequl Islam**: conceptualization, writing – original draft, investigation, methodology, validation, visualization, writing – review and editing, project administration, formal analysis, software, data curation, supervision, resources. All authors contributed to the manuscript, reviewed and approved the final version, and agree to be accountable for all aspects of the work.

## Funding

The authors have nothing to report.

## Ethics Statement

Experimental Design and Technique Were Approved By the GSTU Department of Pharmacy (#gstu‐20PHR035‐01).

## Conflicts of Interest

The authors declare no conflicts of interest.

## Data Availability

The data that support the findings of this study are available from the corresponding author (**Md. Sakib Al Hasan** and **Muhammad Torequl Islam**) upon reasonable request.

## References

[brb371870-bib-0002] Aernout, E. , I. Benradia , J. B. Hazo , et al. 2021. “International Study of the Prevalence and Factors Associated with Insomnia in the General Population.” Sleep Medicine 82: 186–192. 10.1016/j.sleep.2021.03.028. 33957414

[brb371870-bib-0058] Amssayef, A. , I. Bouadid , and M. Eddouks . 2024. “L‐Tartaric Acid Inhibits Diminazene‐induced Vasorelaxation in Isolated Rat Aorta.” Cardiovascular & Hematological Agents in Medicinal Chemistry 22, no. 1: 83–89. 10.2174/1871525721666230406075646. 37038707

[brb371870-bib-0003] Alkattan, A. , E. Alsalameen , and A. Ahmed . 2021. Central Nervous System Depressant Drugs: Updated Review . Preprints.org.

[brb371870-bib-0004] Amssayef, A. , I. Bouadid , and M. Eddouks . 2023. “L‐Tartaric Acid Exhibits Antihypertensive and Vasorelaxant Effects: The Possible Role of eNOS/NO/cGMP Pathways.” Cardiovascular & Hematological Agents in Medicinal Chemistry 21, no. 3: 202–212. 10.2174/1871525721666230111150501. 36635922

[brb371870-bib-0005] Amssayef, A. , I. Bouadid , and M. Eddouks . 2023. “L‐Tartaric Acid Inhibits Diminazene‐Induced Vasorelaxation in Isolated Rat Aorta.” Cardiovascular & Hematological Agents in Medicinal Chemistry 22, no. 1: 83–89. 10.2174/1871525721666230406075646. 37038707

[brb371870-bib-0006] Amssayef, A. , and M. Eddouks . 2022. “In Vivo Antihyperglycemic and Antidyslipidemic Effects of L‐Tartaric Acid.” Cardiovascular & Hematological Disorders Drug Targets 22, no. 3: 185–198. 10.2174/1871529;23666221202091848. 36464866

[brb371870-bib-0007] Bassetti, D. 2022. “Keeping the Balance: GABA_B_ Receptors in the Developing Brain and Beyond.” Brain Sciences 12, no. 4: 419. 10.3390/brainsci12040419. PMC9031223 35447949

[brb371870-bib-0008] Benarroch, E. E. 2012. “GABAB Receptors: Structure, Functions, and Clinical Implications.” Neurology 78, no. 8: 578–584. 10.1212/WNL.0b013e318247cd03. 22351795

[brb371870-bib-0009] Bhuia, M. S. , M. S. Al Hasan , R. Chowdhury , S. A. Ansari , I. A. Ansari , and M. T. Islam . 2024. “Trans‐Ferulic Acid Reduces the Sedative Activity of Diazepam in Thiopental Sodium‐Induced Sleeping Mice: A Potential GABAergic Transmission.” Neurotoxicology and Teratology 106: 107403. 10.1016/j.ntt.2024.107403. 39547315

[brb371870-bib-0010] Blednov, Y. A. , S. Jung , H. Alva , et al. 2003. “Deletion of the α1 or β2 Subunit of GABAAReceptors Reduces Actions of Alcohol and Other Drugs.” The Journal of Pharmacology and Experimental Therapeutics 304, no. 1: 30–36. 10.1124/jpet.102.042960 12490572

[brb371870-bib-0011] Brohan, J. , and B. G. Goudra . 2017. “The Role of GABA Receptor Agonists in Anesthesia and Sedation.” CNS Drugs 31, no. 10: 845–856. 10.1007/s40263-017-0463-7. 29039138

[brb371870-bib-0012] Carbone, E. A. , G. Menculini , R. de Filippis Jr. , et al. 2023. “Sleep Disturbances in Generalized Anxiety Disorder: The Role of Calcium Homeostasis Imbalance.” International Journal of Environmental Research and Public Health 20, no. 5: 4431. 10.3390/ijerph20054431. PMC10002427 36901441

[brb371870-bib-0013] Chaachouay, N. , and L. Zidane . 2024. “Plant‐Derived Natural Products: A Source for Drug Discovery and Development.” Drugs and Drug Candidates 3, no. 1: 184–207.

[brb371870-bib-0014] Cho, S. , and M. Shimizu . 2015. “Natural Sleep Aids and Polyphenols as Treatments for Insomnia.” In Bioactive Nutraceuticals and Dietary Supplements in Neurological and Brain Disease, 141–151. Academic Press. 10.1016/B978-0-12-411462-3.00015-1.

[brb371870-bib-0015] Cholet, C. , S. Claverol , O. Claisse , et al. 2016. “Tartaric Acid Pathways in *Vitis vinifera* L. (cv. Ugni Blanc): A Comparative Study of Two Vintages With Contrasted Climatic Conditions.” BMC Plant Biology 16, no. 1: 144. 10.1186/s12870-016-0833-1. PMC4924324 27350040

[brb371870-bib-0016] Edinoff, A. N. , C. A. Nix , J. Hollier , et al. 2021. “Benzodiazepines: Uses, Dangers, and Clinical Considerations.” Neurology International 13, no. 4: 594–607. 10.3390/neurolint13040059. PMC8629021 34842811

[brb371870-bib-0017] Feng, Y. , Y. Yan , J. He , H. Tao , Q. Wu , and S. Y. Huang . 2022. “Docking and Scoring for Nucleic Acid‐Ligand Interactions: Principles and Current Status.” Drug Discovery Today 27, no. 3: 838–847. 10.1016/j.drudis.2021.10.013. 34718205

[brb371870-bib-0018] Ferini‐Strambi, L. , R. Auer , B. Bjorvatn , et al. 2021. “Insomnia Disorder: Clinical and Research Challenges for the 21st Century.” European Journal of Neurology 28, no. 7: 2156–2167. 10.1111/ene.14784. 33619858

[brb371870-bib-0019] Gulias‐Cañizo, R. , E. D. Ruíz‐Leyja , H. Sánchez‐Castillo , J. Parodí , and L. D. O. de la Paz . 2021. “The Role of GABA Neurotransmitter in the Human Central Nervous System, Physiology, and Pathophysiology.” Revista mexicana de neurociencia 22, no. 2: 67–76. 10.24875/rmn.20000050.

[brb371870-bib-0020] Gupta, R. , S. Grover , A. Basu , et al. 2020. “Changes in Sleep Pattern and Sleep Quality during COVID‐19 Lockdown.” Indian Journal of Psychiatry 62, no. 4: 370–378. 10.4103/psychiatry.IndianJPsychiatry_523_20 PMC7597722 33165382

[brb371870-bib-0021] Ha, J. H. , and A. S. Basile . 1996. “Modulation of Ligand Binding to Components of the GABAA Receptor Complex by Ammonia: Implications for the Pathogenesis of Hyperammonemic Syndromes.” Brain Research 720, no. 1–2: 35–44. 10.1016/0006-8993(96)00104-7 8782894

[brb371870-bib-0022] Harrison, N. L. 2007. “Mechanisms of Sleep Induction by GABA(A) Receptor Agonists.” The Journal of Clinical Psychiatry 68: 6–12. 17539703

[brb371870-bib-0023] Hassan, S. H. , H. A. S. El‐Nashar , M. A. Rahman , et al. 2024. “Sclareol Antagonizes the Sedative Effect of Diazepam in Thiopental Sodium‐Induced Sleeping Animals: In Vivo and in Silico Studies.” Biomedicine & Pharmacotherapy 176: 116939. 10.1016/j.biopha.2024.116939. 38870629

[brb371870-bib-0024] Hosseini, A. , L. Mobasheri , H. Rakhshandeh , V. B. Rahimi , Z. Najafi , and V. R. Askari . 2024. “Edible Herbal Medicines as an Alternative to Common Medication for Sleep Disorders: A Review Article.” Current Neuropharmacology 22, no. 7: 1205–1232. 10.2174/1570159;21666230621143944. PMC10964091 37345244

[brb371870-bib-0025] Kaźmierczyk, I. , M. Bychowski , J. Kwaśna , et al. 2024. “Passiflora Incarnata as an Adjunctive Treatment for Anxiety and Sleep Disorders.” Quality in Sport 35: 56361–56361.

[brb371870-bib-0026] Knoflach, F. , M. C. Hernandez , and D. Bertrand . 2016. “GABAA Receptor‐Mediated Neurotransmission: Not So Simple After All.” Biochemical Pharmacology 115: 10–17. 10.1016/j.bcp.2016.03.014. 27002180

[brb371870-bib-0027] Kousar, M. , U. Salma , T. Khan , and A. J. Shah . 2022. “Antihypertensive Potential of Tartaric Acid and Exploration of Underlying Mechanistic Pathways.” Dose‐Response: A Publication of International Hormesis Society 20, no. 4: 1–10. 10.1177/15593258221135728. PMC9597030 36311175

[brb371870-bib-0028] Küpeli Akkol, E. , I. Tatlı Çankaya , G. Şeker Karatoprak , E. Carpar , E. Sobarzo‐Sánchez , and R. Capasso . 2021. “Natural Compounds as Medical Strategies in the Prevention and Treatment of Psychiatric Disorders Seen in Neurological Diseases.” Frontiers in Pharmacology 12: 669638. 10.3389/fphar.2021.669638. PMC8155682 34054540

[brb371870-bib-0029] Kuriyama, K. , and S. Tomono . 1986. “Significance of the Enhancement of GABA Receptor Binding With Low Affinity in the Assessment of Central Effects of Benzodiazepine Derivatives: Analysis Using a 1H‐1,2,4‐Triazolyl Benzophenone Derivative (450191‐S).” Neurochemistry International 9, no. 1: 91–98. 10.1016/0197-0186(86)90036-7. 20493105

[brb371870-bib-0030] Liu, Z. , and S. Song . 2025. “Effects of Anesthetics on the Central Nervous System.” In Neuroscience of Anesthesia: From Cellular Mechanisms to Clinical Applications, 63–86. Springer Nature Switzerland.

[brb371870-bib-0031] McGowan, N. M. , D. S. Kim , M. de Andres Crespo , L. Bisdounis , S. D. Kyle , and K. E. A. Saunders . 2022. “Hypnotic and Melatonin/Melatonin‐Receptor Agonist Treatment in Bipolar Disorder: A Systematic Review and Meta‐Analysis.” CNS Drugs 36, no. 4: 345–363. 10.1007/s40263-022-00911-7. 35305257

[brb371870-bib-0032] Mednikova, Y. S. , M. K. Kozlov , and A. N. Makarenko . 2019. “Energy Aspects of Sodium Thiopental Action on Nervous Activity.” Journal of Behavioral and Brain Science 9, no. 2: 33–53. 10.4236/jbbs.2019.92004.

[brb371870-bib-0033] Mihic, S. J. , and R. A. Harris . 2011. Hypnotics and Sedatives. Goodman and Gilman's Pharmacological Basis of Therapeutics. 12th ed. McGraw‐Hill, 457–480.

[brb371870-bib-0034] Haneef, M. M. M. , and F. N. Khan . 2025. “Tandem Synthesis of Piriqualone and Analogues Using Deep Eutectic Solvents: Photophysical and Theoretical Insights.” Asian Journal of Organic Chemistry 14, no. 1: e202400487.

[brb371870-bib-0035] Nhlapho, S. , M. H. L. Nyathi , B. L. Ngwenya , et al. 2024. “Druggability of Pharmaceutical Compounds Using Lipinski Rules With Machine Learning.” Sciences of Pharmacy 3, no. 4: 177–192. 10.58920/sciphar0304264.

[brb371870-bib-0036] Nordin, M. , and S. Nordin . 2016. “Sleep and Sleepiness in Environmental Intolerances: A Population‐Based Study.” Sleep Medicine 24: 1–9. 10.1016/j.sleep.2016.06.014. 27810173

[brb371870-bib-0037] Oishi, Y. , Y. C. Saito , and T. Sakurai . 2023. “GABAergic Modulation of Sleep‐Wake States.” Pharmacology & Therapeutics 249: 108505. 10.1016/j.pharmthera.2023.108505. 37541595

[brb371870-bib-0038] Olfson, M. , M. Wall , S. M. Liu , C. M. Morin , and C. Blanco . 2018. “Insomnia and Impaired Quality of Life in the United States.” The Journal of Clinical Psychiatry 79, no. 5: 17m12020. 10.4088/JCP.17m12020. 30256547

[brb371870-bib-0039] Olsen, R. W. 2018. “GABAA Receptor: Positive and Negative Allosteric Modulators.” Neuropharmacology 136: 10–22. 10.1016/j.neuropharm.2018.01.036. PMC6027637 29407219

[brb371870-bib-0040] Oxman, A. D. , S. Flottorp , K. Håvelsrud , et al. 2007. “A Televised, Web‐Based Randomised Trial of an Herbal Remedy (Valerian) for Insomnia.” PLoS One 2, no. 10: e1040. 10.1371/journal.pone.0001040. PMC2002515 17940604

[brb371870-bib-0041] Pádua‐Reis, M. , D. A. Nôga , A. B. L. Tort , and M. Blunder . 2021. “Diazepam Causes Sedative Rather than Anxiolytic Effects in C57BL/6J Mice.” Scientific Reports 11, no. 1: 9335. 10.1038/s41598-021-88599-5. PMC8085115 33927265

[brb371870-bib-0042] Pérez Santín, E. , R. R. Solana , M. González García , et al. 2021. “Toxicity Prediction Based on Artificial Intelligence: A Multidisciplinary Overview.” Wiley Interdisciplinary Reviews: Computational Molecular Science 11, no. 5: e1516. 10.1002/wcms.1516.

[brb371870-bib-0043] Philip, A. B. , J. Brohan , and B. Goudra . 2025. “The Role of GABA Receptors in Anesthesia and Sedation: An Updated Review.” CNS Drugs 39, no. 1: 39–54. 10.1007/s40263-024-01128-6 PMC11695389 39465449

[brb371870-bib-0044] Reynolds, D. S. , T. W. Rosahl , J. Cirone , et al. 2003. “Sedation and Anesthesia Mediated by Distinct GABAA Receptor Isoforms.” The Journal of Neuroscience 23, no. 24: 8608–8617. 10.1523/JNEUROSCI.23-24-08608.2003 PMC6740367 13679430

[brb371870-bib-0045] Riemann, D. , F. Benz , R. J. Dressle , et al. 2022. “Insomnia Disorder: State of the Science and Challenges for the Future.” Journal of Sleep Research 31, no. 4: e13604. 10.1111/jsr.13604. 35460140

[brb371870-bib-0046] Santos, A. L. S. , F. P. Matteoli , L. S. Sangenito , M. H. Branquinha , B. A. Cotrim , and G. O. Resende . 2018. “Asymmetric Peptidomimetics Containing L‐Tartaric Acid Core Inhibit the Aspartyl Peptidase Activity and Growth of Leishmania Amazonensis Promastigotes.” Acta Parasitologica 63, no. 1: 114–124. 10.1515/ap-2018-0013. 29351078

[brb371870-bib-0047] Shinomiya, K. , T. Inoue , Y. Utsu , et al. 2005. “Effects of Kava‐Kava Extract on the Sleep‐Wake Cycle in Sleep‐Disturbed Rats.” Psychopharmacology 180, no. 3: 564–569. 10.1007/s00213-005-2196-4. 15700178

[brb371870-bib-0048] Stielow, M. , A. Witczyńska , N. Kubryń , Ł. Fijałkowski , J. Nowaczyk , and A. Nowaczyk . 2023. “The Bioavailability of Drugs‐The Current State of Knowledge.” Molecules 28, no. 24: 8038. 10.3390/molecules28248038. PMC10745386 38138529

[brb371870-bib-0049] Strähl, A. N. 2023. “Insomnia in Modern World.” Doctoral diss., University of Zagreb. School of Medicine.

[brb371870-bib-0050] Sucharitha, P. , K. R. Reddy , S. V. Satyanarayana , and T. Garg . 2022. “Absorption, Distribution, Metabolism, Excretion, and Toxicity Assessment of Drugs Using Computational Tools.” In Computational Approaches for Novel Therapeutic and Diagnostic Designing to Mitigate SARS‐CoV‐2 Infection, 335–355. Academic Press. 10.1016/B978-0-323-91172-6.00012-1.

[brb371870-bib-0051] Sultana, T. , M. A. Mannan , and T. Ahmed . 2018. “Evaluation of central Nervous System (CNS) Depressant Activity of Methanolic Extract of Commelina Diffusa Burm. in Mice.” Clinical Phytoscience 4, no. 1: 5.

[brb371870-bib-0052] Tang, S. C. , and J. H. Yang . 2018. “Dual Effects of Alpha.‐Hydroxy Acids on the Skin.” Molecules 23, no. 4: 863. 10.3390/molecules23040863. PMC6017965 29642579

[brb371870-bib-0053] Varinthra, P. , S. N. M. N. Anwar , S. C. Shih , and I. Y. Liu . 2024. “The Role of the GABAergic System on Insomnia.” Tzu chi Medical Journal 36, no. 2: 103–109. 10.4103/tcmj.tcmj_243_23. PMC11025592 38645778

[brb371870-bib-0054] Veena, N. S. , K. Sivaji , G. V. Benerji , M. F. Babu , and D. R. Kumari . 2015. “Skeletal Muscle Relaxant Property of Diazepam by Using Rotarod on Albino Mice.” Indian Journal of Basic and Applied Medical Research 4, no. 4: 714–721.

[brb371870-bib-0055] Verma, S. , and R. K. Pathak . 2022. “Discovery and Optimization of Lead Molecules in Drug Designing.” In Bioinformatics, 253–267. Academic Press. 10.1016/B978-0-323-89775-4.00004-3.

[brb371870-bib-0056] Yeh, C. H. , C. K. Arnold , Y. H. Chen , and J. N. Lai . 2011. “Suan zao ren tang as an Original Treatment for Sleep Difficulty in Climacteric Women: A Prospective Clinical Observation.” Evidence‐Based Complementary and Alternative Medicine: eCAM 2011: 673813. 10.1155/2011/673813. PMC3108138 21660310

[brb371870-bib-0057] Zhao, Y. , Y. Wu , and M. Wang . 2014. “Bioactive Substances of Plant Origin.” In Handbook of Food Chemistry, 1–35. Springer. 10.1007/978-3-642-36605-5_13.

